# Quality by Design Applied Development of Immediate-Release Rabeprazole Sodium Dry-Coated Tablet

**DOI:** 10.3390/pharmaceutics13020259

**Published:** 2021-02-14

**Authors:** Sang-Ho Lee, Joo-Eun Kim

**Affiliations:** Department of Pharmaceutical Engineering, Catholic University of Daegu, Hayang-Ro 13-13, Gyeongsan City 38430, Gyeongbuk, Korea; wws7316@naver.com

**Keywords:** quality by design, proton pump inhibitor, rabeprazole sodium, sodium bicarbonate, immediate release, pharmacokinetics

## Abstract

The aim of this study was to develop immediate-release oral rabeprazole sodium tablets with rapid efficacy and gastric stability for the treatment of gastroesophageal reflux disease. Rabeprazole sodium is a commonly prescribed proton pump inhibitor; however, it is extremely unstable and degrades in acidic environments. Hence, it has been manufactured and supplied only in enteric-coated tablet form, while immediate-release (IR) formulations for this drug are very limited. In this study, we applied the quality by design (QbD) approach to formulate and optimize an IR dry-coated tablet containing rabeprazole sodium as an inner core with an outer sodium bicarbonate layer to stabilize the active pharmaceutical ingredient at gastric pH. We also investigated the stability of the pharmaceutical dosage form and its pharmacokinetic profile. The results show that the developed tablets are stable for approximately 12 months and have a high dissolution rate, greater than or equal to 90% at 30 min. Further, in vivo beagle pharmacokinetics confirmed that the newly developed IR tablet had an AUC_t_ which is bioequivalent to the existing delayed-release rabeprazole tablet; however, its T_max_ was 0.5 h, which is up to seven times faster than that of the existing tablet. Moreover, the IR tablet was found to immediately absorb in the stomach. Hence, the development of IR tablets can be used as a platform to overcome the technical and commercial limitations currently associated with various proton pump inhibitors used to treat patients with gastroesophageal reflux disease that require immediate therapeutic relief.

## 1. Introduction

Proton pump inhibitors (PPIs) irreversibly block H^+^-ATPase and K^+^-ATPase involved in the final stage of gastric acid secretion. PPIs achieve this by passing through the parietal basement membrane and accumulating in the secretory canaliculus, where they become activated when the gastric acid pH is <4.0. They are then converted to the sulfenamide form and covalently bind the cysteine group in the proton pump, thereby irreversibly inhibiting acid secretion [1,2]. Although gastroesophageal reflux disease (GERD) is commonly treated with various drugs, including H2RA and P-CAB, PPIs are considered the most effective drug therapy for GERD [3]. PPIs are benzimidazole derivatives, of which rabeprazole, omeprazole, and esomeprazole are the most frequently prescribed [4]. However, unlike other PPIs, rabeprazole sodium is not affected by CYP2C19 genetic polymorphism [5,6,7], making it relatively less likely to interact with other drugs [8,9,10]. Nevertheless, rabeprazole sodium readily decomposes at acidic and neutral pH ≤ 7.0. Specifically, the decomposition half-life of rabeprazole sodium is <10 min in aqueous solutions with pH < 3.0. Rabeprazole sodium is also adversely affected by moisture, heat, organic solvents, and light [11,12]. Therefore, it is challenging to formulate conventional rabeprazole sodium tablets that are physicochemically stable. In addition, as traditional rabeprazole sodium formulations contain large amounts of alkali stabilizers and various excipients, they have the potential to induce problems with stability and low quality. For these reasons, rabeprazole sodium is currently prepared in enteric-coated tablets and delayed-release capsules to avoid decomposition in low gastric pH. However, the enteric-coated tablets and delayed-release capsules were designed to dissolve and be absorbed in the intestine without being immediately absorbed in the stomach. Therefore, the drug onset time is long and deemed unsuitable for the treatment of GERD requiring immediate therapeutic efficacy [13,14]. Generally, enteric-coated rabeprazole tablets (Pariet^®^) have a very slow pharmacokinetic onset time, with a T_max_ = 3.5–4.5 h [15,16,17,18]. Hence, within the pharmaceutical industry, there is a general consensus that, to adequately and effectively alleviate the pain associated with GERD, the development of rapid absorption PPI formulas with good safety profiles is required [19,20,21]. However, this challenge has not yet been met due to the unstable physicochemical properties associated with PPI drugs.

However, until recently, no studies have reported immediate-release PPI formulations. This is largely due to its poor stability leading to rapid decomposition, making it difficult to manufacture as a fast-acting agent [22]. Moreover, past formulations have been focused on avoiding the gastric pH range in the Gastrointestinal (GI) tract rather than that of the stomach [23]. In previous studies, gastric pH was raised using an antacid, such as sodium bicarbonate [24,25] or calcium carbonate [6,26]; however, these agents were not used in combination with PPI.

The purpose of the current study is, therefore, to design a stable, immediate-release (IR) PPI drug that will rapidly absorb into the stomach, while ensuring similar safety, stability, and efficacy as that associated with traditional rabeprazole sodium formulations. Specifically, a research goal was to make the time to maximum concentration (T_max_) > 3× shorter than those of the existing delayed-release formulations. To this end, we sought to neutralize gastric acid using an antacid, such as sodium bicarbonate, to allow for the immediate release of PPI into the stomach, thereby inducing a faster onset time within one hour compared to conventional rabeprazole sodium [27]. Nevertheless, challenges in the research and development process were anticipated. We designed a formulation by applying the quality by design (QbD) approach and attempted to optimize it via statistical formulation design evaluation of the design of experiment (DoE) [28]. To this end, we mapped the material attributed (MA) and the process parameters (PPs), based on a risk assessment (RA). We then selected the critical material attributes (CMAs) and critical process parameters (CPPs) affecting formulation design. The ranges of the selected CMAs and CPPs were optimized with a central composite design among the experimental methods (DoE). 

In preliminary studies, we observed that sodium bicarbonate stabilized rabeprazole sodium, leading us to design a monolayer IR tablet with sodium bicarbonate and rabeprazole sodium. However, contact between the sodium bicarbonate monolayer and the inner rabeprazole core disrupted the stability of the tablet, resulting in breakdown within four weeks, which falls short of the common 24-month shelf life for drugs. Therefore, we developed rabeprazole sodium-sodium bicarbonate complex dry-coated tablets (RS dry-coated tablets) containing an inner core of rabeprazole sodium and an outer layer of sodium bicarbonate that was stabilized by preventing contact between the active pharmaceutical ingredients (APIs) via double coating of the inner core. Hence, the results of this study provide theoretical evidence that rabeprazole stability could be realized via the neutralization of gastric acid by sodium bicarbonate. Moreover, the development of IR tablets represents a potential for the design of PPI drugs to treat patients with GERD requiring immediate therapeutic relief. 

## 2. Materials and Methods

### 2.1. Materials

The active pharmaceutical ingredient (API) rabeprazole sodium was purchased from Ildong (Seoul, Korea), while sodium bicarbonate was purchased from Hebei Huachen Pharmaceutical Co. Ltd. (Huanghua, Hebei, China). Additionally, the following reagents were used throughout the study: *D*-mannitol (Roquette, Lestrem, France), heavy calcium carbonate (Shanghai Nuocheng Pharmaceutical Co. Ltd., Shanghai, China), magnesium oxide (Tomita Pharmaceutical Co. Ltd., Tokushima, Japan), calcium hydroxide (Spectrum Pharmaceuticals, Henderson, NV, USA), hydroxypropyl cellulose (Nippon Soda Co. Ltd., Tokyo, Japan), sodium starch glycolate (JRS Pharma, Rosenberg, Germany), low-substituted hydroxypropyl cellulose and ethylcellulose (Ashland, Covington, KY, USA), magnesium stearate (Faci Asia Pacific Pty Ltd., Jurong Island, Singapore), talc (Nippon Talc Co. Ltd., Osaka, Japan), titanium dioxide (Huntsman Corporation, Oulsburg, Germany), aluminum lake yellow No. 4 (Borak, Hwaseong, Gyeonggi, Korea), as well as copovidone and crospovidone (BASF Co. Ltd., Ludwigshafen, Germany). All other chemicals were of analytical reagent grade and purchased commercially.

### 2.2. Physicochemical Properties of APIs

#### 2.2.1. Solubility Studies for Rabeprazole Sodium and Sodium Bicarbonate 

The solubility of each component in deionized water, solvents (ethanol, methanol), citric acid/sodium citrate buffer, and pH buffer was evaluated [29]. To assess the stability of rabeprazole sodium and sodium bicarbonate at various pH, color morphology was observed after solubility test completion for 24 h.

Rabeprazole sodium solubility was evaluated by the apparent and equilibrium solubility test methods and analyzed by HPLC (Waters 1529; UV/Vis 2707; Waters, Milford, MA, USA). Each vial contained 10 mg rabeprazole sodium, and 200 µL of each solvent was slowly added. Dissolution was visually confirmed and apparent drug solubility was calculated from the time when all active ingredients were dissolved and no particles were visible. Excess rabeprazole sodium (400 mg) was then added, and 1 mL aliquots were collected at 1 h, 5 h, and 24 h. The solutions were filtered and then diluted 2000× to measure the quantity of rabeprazole sodium dissolved in the solution. Equilibrium or final rabeprazole sodium solubility was calculated from the HPLC peak.

$$\frac{\mathrm{Peak}\;\mathrm{area}\;\mathrm{of}\;\mathrm{sample}\;\mathrm{solution}\;\times \;100\;\times \;100}{\mathrm{Average}\;\mathrm{of}\;\mathrm{standard}\;\mathrm{solution}\;\mathrm{peak}\;\mathrm{area}\;\times 1000}$$

The concentration of the standard solution = 100 μg/mL.

Sodium bicarbonate could not be analyzed using HPLC and was evaluated via the apparent solubility test method [30]. Each vial contained 10 mg sodium bicarbonate, and 200 μL of each solvent was slowly added. Dissolution was visually confirmed, and apparent drug solubility was calculated from the time when all active ingredients were dissolved and no particles were visible. If the sodium bicarbonate did not dissolve in ≤100 mL, it was deemed nearly insoluble (Solubility Study Method <1236> in the U.S. Pharmacopeia) [31].

#### 2.2.2. Compatibility Studies for the Selection of APIs and Excipients

To confirm compatibility between the APIs, rabeprazole sodium and sodium bicarbonate were mixed at 1:1 and 20:800 (*w*/*w*) ratios. The latter reflects the proportions of APIs in each commercialized tablet. The prepared samples were stored in a stability chamber for 4 weeks at room temperature of 25 ± 2 °C/ 60 ± 5% RH and under acceleration conditions of 40 ± 2 °C/ 75 ± 5% RH, respectively. Interaction between rabeprazole sodium and sodium bicarbonate was confirmed using reported impurities in USP43-NF38 rabeprazole sodium monographs and monitored by HPLC (Agilent 1260 Infinity II; Agilent Technologies, Santa Clara, CA, USA). To confirm interactions and compatibility between rabeprazole sodium and various excipients, differential scanning calorimetry (DSC Q2000; TA Instruments, Newcastle, DE, USA) and HPLC (Agilent 1260 Infinity II; Agilent Technologies, Santa Clara, CA, USA) were conducted. Rabeprazole sodium was mixed at a 1:1 ratio (*w*/*w*) with the following excipients: mannitol, lactose, dicalcium phosphate dihydrate, microcrystalline cellulose, pregelatinized starch, precipitated calcium carbonate, sodium starch glycolate, crospovidone, sodium croscarmellose, sodium stearyl fumarate, magnesium stearate, povidone K, hydroxypropyl methylcellulose, hydroxypropyl cellulose, calcium hydroxide, and sodium hydroxide. Each mixture was stored in a stability chamber for 4 weeks at room temperature of 25 ± 2 °C/ 60 ± 5% RH and under acceleration conditions of 40 ± 2 °C/ 75 ± 5% RH. After 4 weeks, 1–3 mg of each mixture was heated from 25–330 °C at 10 °C/min and DSC was used to monitor changes in the endothermic and exothermic peaks [32]. If none were detected, an additional impurity test was performed to confirm compatibility. For sodium bicarbonate, neither DSC nor HPLC could be performed, so its compatibility with other excipients was determined from literature searches and prior experience [33,34,35].

### 2.3. Analysis of Acid-Neutralizing Capacity Based Sodium Bicarbonate Dose

The acid-neutralizing capacity by sodium bicarbonate dose was performed according to the acid-neutralizing capacity method <301> in USP Pharmacopeia. The average weight corresponding to the daily dose of sodium bicarbonate (range of 200–1000 mg) was calculated and added to separate 200 mL flasks, to which 100 mL of 0.1 M HCl was added. The solution was then stirred at 37 ± 2 °C for 1 h and filtered by polyvinylidene fluoride (PVDF) membrane filter. Next, 50 mL of the filtrate was obtained and titrated with hydrochloric acid with 0.1 M sodium hydroxide solution (pH measurement method) with an endpoint of pH 3.5. A blank was also generated in the same manner and the acid-neutralizing capacity was calculated as follows:

$$acid-neutralizing\;capacity\;\left(mL\right)=\left(b-a\right)f\;\times 2\;\times \left(\frac{t}{s}\right)$$

where *a* represents the amount of 0.1 M NaOH consumed (mL), *b* is 0.1 M NaOH consumption of the blank test (mL), *f* is the standard coefficient of 0.1 mol/L sodium hydroxide solution (1.0), *t* is the daily dose of sodium bicarbonate, and *s* is the amount of sample (mg).

### 2.4. Quality Target Product Profile (QTPP), Critical Quality Attributes (CQAs), and Risk Assessment of CMAs and CPPs (Preliminary Hazard Analysis (PHA) and Failure Mode Effect Analysis (FMEA))

The quality target product profile (QTPP) is the basis for formulation and production process design in drug development [36,37,38]. Parameters include clinical use, route of administration, formulation, delivery system, content, container and packaging, API release or delivery, characteristics affecting pharmacokinetic properties, sterility, purity, stability, and dissolution (Table 1). The QTPP was justified by evaluating its feasibility. To establish the direction of product and process development, the critical quality attributes (CQAs) of the drugs were identified through QTPP and prior knowledge. To identify the CQAs, physical characteristics, appearance, identification, dissolution, impurities, weight variation, content uniformity, assay, and residual solvents were specified (Table 2). All variables potentially affecting quality were identified via RA. For the RA, preliminary hazard analysis (PHA) and failure mode effect analysis (FMEA) were used and were based on prior knowledge and initial experimental data. The PHA was rated green, yellow, or red according to the degree of risk. Green represented a wide range of acceptable risks and yellow represented acceptable risk. A yellow rating might necessitate additional research and feasibility studies to lower the risk. Red represented unacceptable risk and research is essential to lower the risk. FMEA generates probability-based failure scores (P), detectability based on CQA severity impact (D), and the probability and severity of undetected failures (S). The score was calculated using risk priority number (RPN) and classified as low-impact, medium-impact, or high-impact. Variables with RPN ≥ 30 were defined as a CMA and a CPP. Additional measures were implemented through the DoE to prepare for risk.

### 2.5. Formulation Studies on RS Dry-Coated Tablets

Rabeprazole sodium bicarbonate complex dry-coated tablets (RS dry-coated tablets) were selected and optimized based on the compatibility, QTPP, and CQA results. Stabilizing excipients were selected according to compatibility and preliminary study output. The optimum composition and manufacturing process were selected by adjusting the excipient range through the design space. The inner core tablet was manufactured as previously described [39,40,41].

An inner core was prepared by wet granulation. Rabeprazole 20 mg, *D*-mannitol, precipitated calcium carbonate, magnesium oxide, calcium hydroxide, hydroxypropyl cellulose, sodium starch glycolate, and low-substituted hydroxypropyl cellulose were mixed and an ethanol solution was added as a binder to prepare wet granules. A high-speed mixer (PM-1060; PTK, Gimpo-si, Korea) was used with impeller and chopper speeds of 150 ppm and 1600 rpm, respectively. The run time was 3 min. The granules were dried in a cabinet dryer at 40 °C for ≥3 h until the moisture content was <2%. The dried granules were passed through a 16-mesh sieve and magnesium stearate was added. The inner core tablets were manufactured by compressing them to 5 kp hardness and 150 mg weight in a rotary compression machine (PR-LM Series; PTK, Gimpo-si, Korea). The inner core tablet was completed with a double seal and hydroxypropyl methylcellulose (HPMC) coating and the final weight was 163 mg. The outer tablet layers were manufactured with a rotary dry-coated compression machine (PR-3000 Series; PTK, Gimpo-si, Korea). However, sodium bicarbonate had only weak binding power and might have been detrimental to the quality attributes if it were used in the manufacturing of general compositions. Hence, DoE-mediated optimization and design space derivation were required. Therefore, the central composite design for the main factors was performed and optimized.

### 2.6. In Vitro Dissolution Studies

The tablet dissolution profiles were investigated with a dissolution tester (PTWS 120D^®^; Pharma Test, Hainburg, Germany) using the paddle method (method 2, USP23). The rabeprazole sodium dissolution medium was a pH 8.0 buffer and was selected according to the solubility and stability test results. Sodium bicarbonate dissolution patterns did not vary among media. Thus, water was selected as the sodium bicarbonate dissolution medium. The tablets were placed in dissolution media heated to 37 ± 0.5 °C and dissolution was conducted for 45 min. According to the paddle method of the USP<711>, rabeprazole sodium was evaluated for 5, 10, 15, 30, and 45 min at 75 rpm, and sodium bicarbonate was evaluated for 5, 10, 15, 30, and 45 min at 75 rpm, both at 37.0 ± 0.5 °C. Samples were collected and passed through the regenerated cellulose (RC) filter (0.45 µm; 25 mm). The rabeprazole sodium samples were then pretreated with 0.5 M NaOH and passed through the RC filter. The concentrations of APIs in each sample were determined by HPLC and ion chromatography [42].

### 2.7. Stability Studies

RS dry-coated tablets and the reference drug were stored in a stability chamber at 25 ± 2 °C and 60 ± 5% RH for 12 months. Assay, content uniformity, dissolution, impurities, and stability were evaluated (Pharmaceutical Stability Method <1150> in the USP Pharmacopeia) [43]. Content uniformity was tested for ten samples of each API according to the USP test method. Dissolution was tested using the dissolution profile method. Rabeprazole sodium impurities were identified according to the criteria established in International Council for Harmonisation (ICH) Guideline No. Q3. RS dry-coated tablet shelf life was evaluated using the storage/stability test.

### 2.8. Analytical Methods

#### 2.8.1. HPLC

Rabeprazole sodium assay was performed, and impurities were analyzed using an HPLC (Agilent 1260 Infinity II; Agilent Technologies, Santa Clara, CA, USA) apparatus fitted with a separation module and a UV detector. For the rabeprazole sodium assay, Inertsil octa-decyl silica (ODS) (4.6 mm × 150 mm; 5 µm) served as the stationary phase and phosphate buffer (pH 7.0) solution was the mobile phase [44]. The column temperature, flow rate, injection volume, and detector were 30 °C, 1.2 mL/min, 10 µL, and 290 nm, respectively. The rabeprazole sodium content uniformity and dissolution tests were performed under the same conditions as those for the chromatographic assay. Phenomenex C18 (4.6 mm × 250 mm; 5 µm) served as the stationary phase for the analysis of rabeprazole sodium impurities. The mobile phase was analyzed using methanol, acetonitrile (ACN), and phosphate buffer (pH 7.0) gradients. The column temperature, flow rate, injection volume, and detector were 45 °C, 1.0 mL/min, 5 µL, and 280 nm, respectively [45,46].

#### 2.8.2. Ion Chromatography

The sodium bicarbonate assay was conducted in an ion chromatography system fitted with a separate module and a conductivity detector. The stationary phase was Metrosep A Supp 4-250/4.0 Metrohm, and the mobile phase was a mixture of 4-hydroxybenzoic acid and 2-(diethylamino)ethanol. The column temperature, flow rate, injection volume, and detector were 25 °C, 1.5 mL/min, 50 µL, and Polarity+, respectively. The sodium bicarbonate weight variation and dissolution tests were performed under the same conditions as those for the chromatographic assay [47].

### 2.9. Pharmacokinetic Studies

The pharmacokinetic profiles of the RS dry-coated tablet and the reference tablet (Pariet^®^) were determined for ≥12 male beagles aged 8–12 months and weighing 9.4–10.5 kg. They were provided by QuBEST BIO Inc. (Gyeonggi, Seongnam, Korea). The dogs were randomly divided into the reference drug and the RS dry-coated tablet groups and subjected to 2 h cross-oral administration. The optimized formulation was administered orally and 10 mL water was supplied after dosing. Approximately 500 µL of blood was collected in heparin tubes at 0.083 h, 0.25 h, 0.5 h, 0.75 h, 1 h, 1.5 h, 2 h, 3 h, 4 h, 6 h, 12 h, and 24 h after administration. The plasma was separated by centrifugation at 12,000 rpm and 4 °C for 2 min. The rabeprazole sodium concentration in the plasma was determined by normalized LC-MS/MS (QTRAP^®^ 4500; AB Sciex LLC, Framingham, MA, USA). All animal testing procedures complied with the Animal Welfare Act and the Guidelines for the Protection and Use of Laboratory Animals and were approved by the Animal Care and Use Committee of QuBEST BIO Inc, Korea (No. KNOTUS-IACUC-20-KE-102, 13 March 2020) [48].

### 2.10. Statistical Analysis

Data were expressed as means ± SD. Treatment means were compared by one-way ANOVA and pairwise comparisons were made by the least significant difference (LSD) test. Differences were considered statistically significant when *p* ≤ 0.05. Minitab^®^ v. 18 (Minitab Inc., University Park, PA, USA) was used for all statistical analyses. Pharmacokinetic parameters were calculated by noncompartmental analysis in Phoenix^®^ WinNonlin^®^ v. 8.2 (Pharsight, Mountain View, CA, USA). The area under the curve (AUC) from time zero until the last measured concentration (AUC_0–t_) was calculated by the trapezoidal method. The AUC from time zero to infinity (AUC_0–∞_) was AUC_0–t_ and the last measured plasma concentration vs. the clearance rate and was calculated as a constant ratio. C_max_ was calculated as the measured maximum plasma concentration. T_max_ was calculated as the time to reach C_max_. The pharmacokinetic parameters of the RS dry-coated and Pariet^®^ tablets were also determined. Individual C_max_ and AUC values, as well as their ratios (test/reference), were compared by logarithmic transformation. Means and 90% confidence intervals (CI) were calculated by parametric ANOVA. In the crossover design, two-way ANOVA was used to evaluate the effects of formulation, duration, and order of administration on pharmacokinetic parameters as a fixed effect and the effects of subjects nested within the sequence as random effects. As a result of the nature of the normal theoretical CI, the analysis was equivalent to performing two one-sided *t*-tests at the 5% significance level.

## 3. Results and Discussion

### 3.1. Physicochemical Properties of APIs

#### 3.1.1. Solubility Studies for Rabeprazole Sodium and Sodium Bicarbonate

The secondary objective of the solubility studies was to measure the solubility of rabeprazole sodium and sodium bicarbonate in each buffer, while the primary objective was to determine the changes to rabeprazole sodium content (%) in various pH, which is reflective of the drugs stability. The objective for measuring drug solubility relates to its importance as an indicator for its absorption patterns in the human body and the design of the tablet formulation (Table 3).

**Figure 1:**
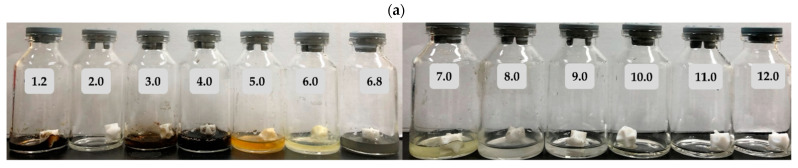

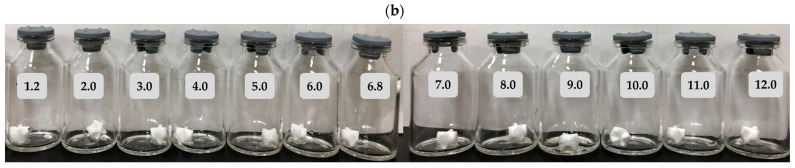
Changes in the appearance of (**a**) rabeprazole sodium and (**b**) sodium bicarbonate in the range of pH 1.2–12.0 (apparent solubility test method).

Figure 1a shows that the relative appearance of the rabeprazole sodium changed in buffers with pH < 7.0, such as the Maillard reaction. In contrast, rabeprazole sodium was transparent in buffers with pH > 8.0. In fact, when rabeprazole sodium was measured according to the pH, it was found to be as low as about 30 to 60% at pH 1.2 to 6.0. Rabeprazole sodium was, therefore, very unstable and degraded at low pH, causing the immediate reaction of the decomposed compound. Therefore, a new method was devised for assessing rabeprazole sodium solubility at pH ≤ 7.0. Rabeprazole sodium solubility was measured by placing excess drug in buffer with pH ≤ 7.0 and subjecting it to pH ≥ 9.0 by adding 0.5 M NaOH. Rabeprazole sodium concentration was >110 mg/mL in all solvents. As the inner core tablet dissolved in general gastric juice, it had adequate solubility to ensure absorption in the human body. Moreover, these conditions were favorable for wet granulation during inner core tablet manufacturing.

Figure 1b shows that the sodium bicarbonate remained transparent and did not change in appearance at any pH. Hence, unlike rabeprazole sodium, sodium bicarbonate remained stable during the pH change. Sodium bicarbonate was highly soluble at pH 6.0, 6.8, 8.0, and 9.0 according to Korean Pharmacopeia criteria. In contrast, it was only slightly soluble in ethanol. Thus, the use of an organic solvent is not appropriate for the manufacturing of the outer layer. It was also confirmed that sodium bicarbonate solubility in ethanol was lower than that in solutions at pH 6.0, 6.8, 8.0, and 9.0, which were all in the pKa range. To evaluate the stability of rabeprazole sodium and sodium bicarbonate solutions at various pH, we observed sample color morphology for 24 h after the solubility test. No significant changes were noted in the color properties for 24 h in solution at pH ≥ 7.0. It was assumed that the onset of PPI action was within ~2 h. When the gastric pH was above neutrality, we assumed there to be no drug degradation or loss in the stomach during that time.

#### 3.1.2. Compatibility Studies for the Selection of APIs and Excipients

Compatibility between APIs was confirmed for the 1:1 (*w*/*w*) and 20:800 (*w*/*w*) mixtures of rabeprazole sodium and sodium bicarbonate (Table 4). Impurity tests were performed on each mixture in the first and fourth weeks. The standard for determining stability was based on ICH guideline Q3 and set to <3.5% of all related substances. Table 1 shows that for measurements for rabeprazole sodium alone, the impurity level was below the 3.5% threshold. In contrast, the impurity level of the 1:1 ratio mixture increased from 0.57% to 0.79% and 2.17%, and that for the 20:800 mixture increased from 3.41% to 6.80% and 9.83%, depending on time and storage conditions. Hence, the formulation was destabilized due to interactions between rabeprazole and sodium bicarbonate. Contact between the APIs in the final formulation might interfere with tablet stability. Therefore, a design that prevents contact between APIs was required to ensure tablet stability.

**Figure 2:**
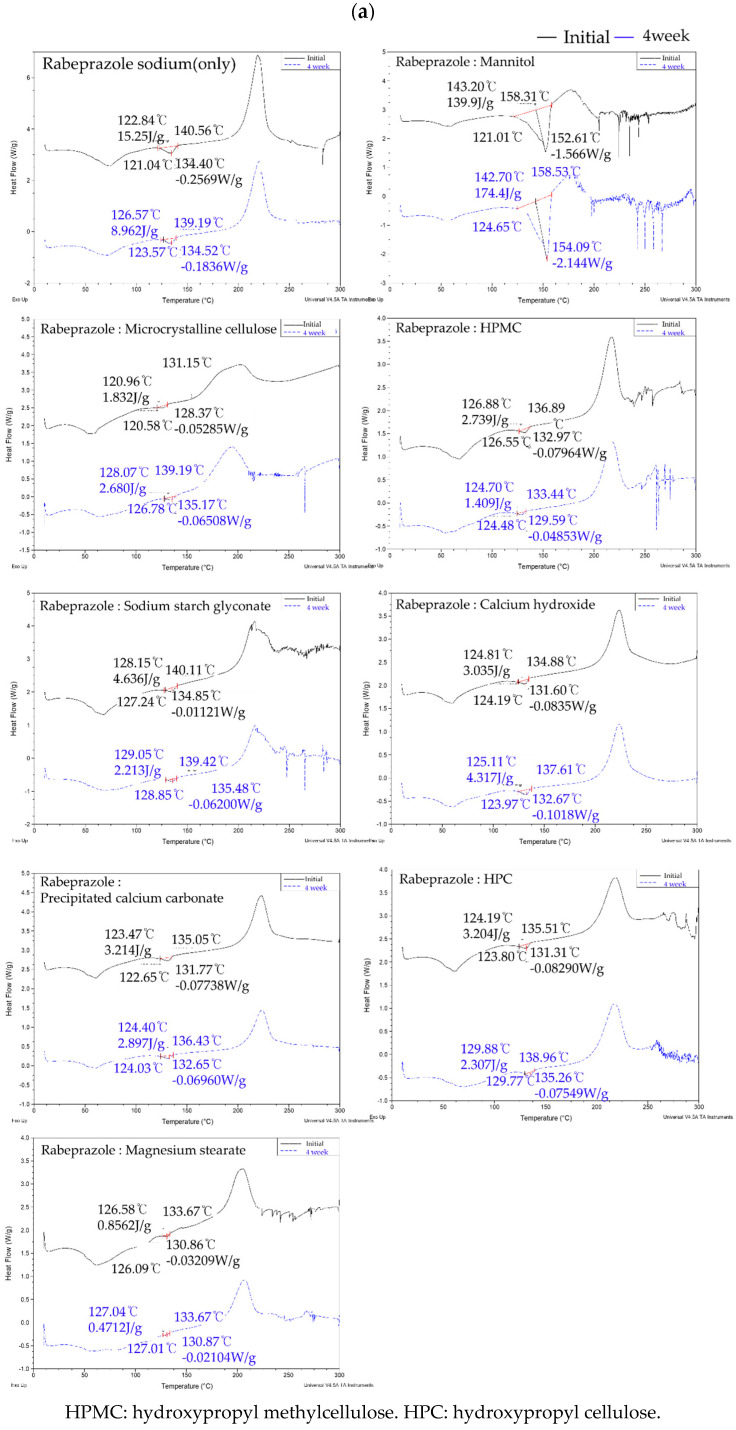

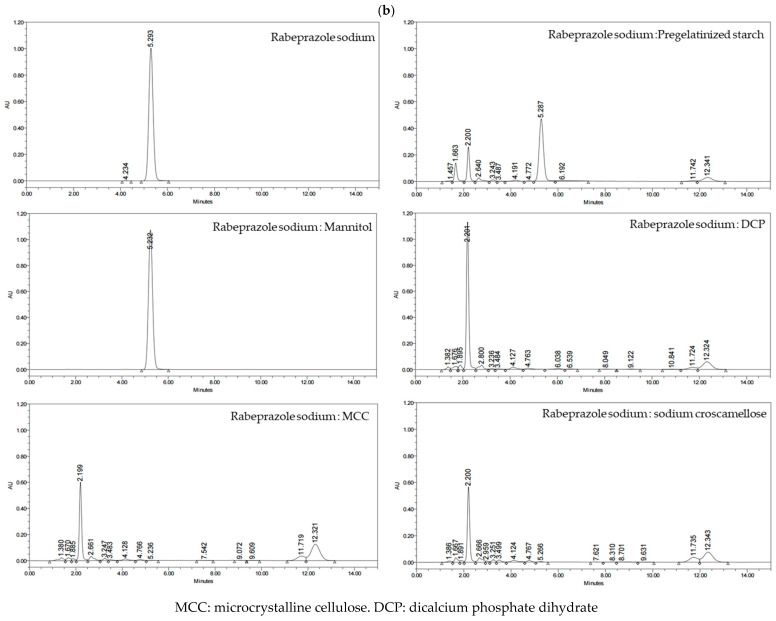
(**a**) Rabeprazole sodium/ each excipient compatibility test via differential scanning calorimetry (DSC). (**b**) Rabeprazole sodium/ each excipient compatibility test via impurities of HPLC.

A DSC analysis was performed on the rabeprazole sodium/excipient mixtures to confirm their mutual compatibility (Figure 2a). The rabeprazole sodium melting point range was 140–141 °C. Figure 2a shows no significant peak shift between room temperature 25 ± 2 °C/ 60 ± 5% RH and the acceleration condition of, 40 ± 2 °C/ 75 ± 5% RH. However, browning occurred in 1:1 mixtures of rabeprazole sodium and dicalcium phosphate dihydrate, croscarmellose sodium, microcrystalline cellulose, or pregelatinized starch because of the Maillard reaction. In these cases, degradation was expected in response to the effects of elevated temperature and humidity. As interactions occurred between components, other impurity tests were conducted (Figure 2b). Chromatographic data for rabeprazole sodium alone and a mixture of rabeprazole sodium and *D*-mannitol confirmed that no decomposition products were formed. For the mixtures of rabeprazole sodium with dicalcium phosphate dihydrate, sodium croscarmellose, microcrystalline cellulose, and pregelatinized starch, wherein the Maillard reaction occurred, the concentration of decomposition products exceeded the 3.5% threshold. Therefore, excipients inducing maillard reactions and causing product decomposition could destabilize the inner core tablet.

The scientific literature and prior experiments revealed that sodium bicarbonate was compatible with copovidone, crospovidone, and magnesium stearate. Therefore, a formulation study was conducted using three excipients with proven stability as binders, disintegrants, and lubricants.

### 3.2. Studies for Acid-Neutralizing Capacity by Sodium Bicarbonate Dose

Through the test for acid-neutralizing capacity, the amount of sodium bicarbonate that rabeprazole sodium can stably dissolve and absorb in the stomach was established. Acid-neutralizing capacity results show that 0.1 M HCl consumption (acid-neutralizing capacity) for 200, 300, 400, 500, 600, 700, 800, 900, and 1000 mg of sodium bicarbonate increased in the order of 38, 42, 45, 49, 57, 68, 79, 84, and 89 mL, respectively. Through a preliminary study, it was confirmed that when the acid-neutralizing capacity is 80 mL or more, the environment of gastric acid can be raised to the level of neutral pH. At the formulation design stage, we set the dosage range for sodium bicarbonate to ensure the stability of rabeprazole sodium without eliciting adverse effects caused by overdose. Therefore, we have set the optimum amount of sodium bicarbonate to 800 mg per tablet to sufficiently neutralize the gastric pH.

### 3.3. QTPP, CQAs, and RA of CMAs and CPPs (PHA and FMEA)

Table 5 and Table 6 show QTPP determination and justification and relative CQA risk classification by color. Appearance, assay, content uniformity, impurities, and dissolution were selected as CQAs. Table 4 and Table 5 show that CMAs and CPPs were selected by PHA and FMEA. The latter two were conducted according to preliminary testing and prior experience. Adequate control of PPs is feasible based on prior investigation and experience. Hence, PPs were not selected as CPPs. The binders and disintegrants in the outer layer were risk factors identified by the RPN score and were to be considered in advance. Up to a limit, tablet hardness increases with binder quantity. Nevertheless, excess binder retards disintegration and increases tablet size, which lowers patient compliance. In contrast, excess disintegrant decreases tablet hardness and interferes with the desired dissolution pattern. Therefore, experimental design metrics are required to establish appropriate ranges for both CMAs. The lubricant RPN score was 27 points, but no corrective measures were taken in the experimental design. Magnesium stearate was tested as a lubricant for the outer layer at an initial 1.0% of the total weight. During mixing, however, tabletability was reduced because of mixture agglomeration. Therefore, tabletability was studied with respect to composition change. It was confirmed that excess lubricant could cause agglomeration and adversely affect tabletability. However, lower lubricant concentrations cause no agglomeration and do not impede tabletability. Therefore, lubricants were not selected as CMAs and the experimental design included a fixed content of 0.2%, which did not hinder tabletability.

### 3.4. Formulation Studies

**Figure 3:**
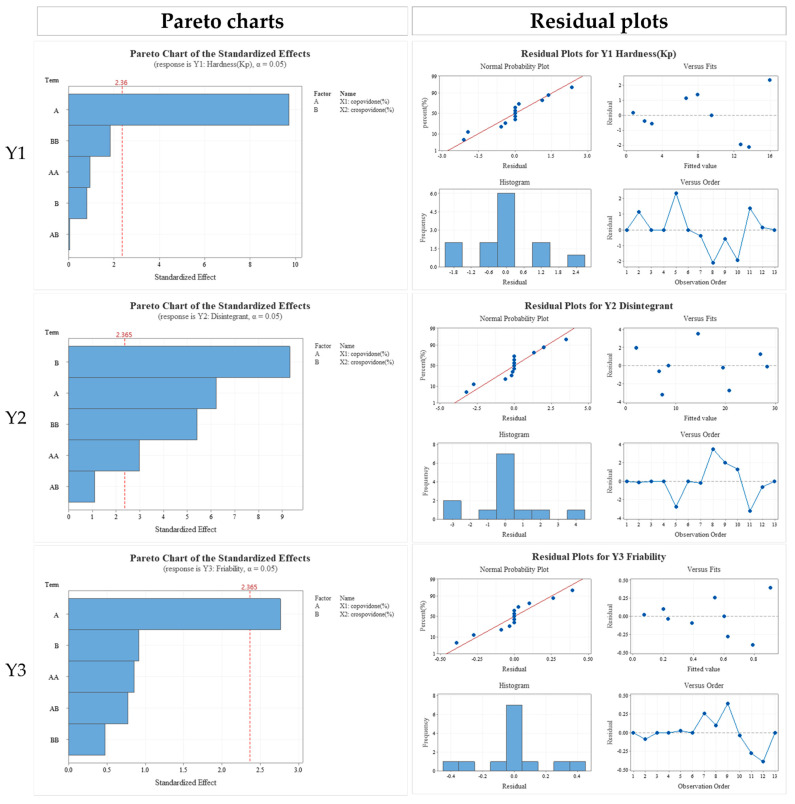

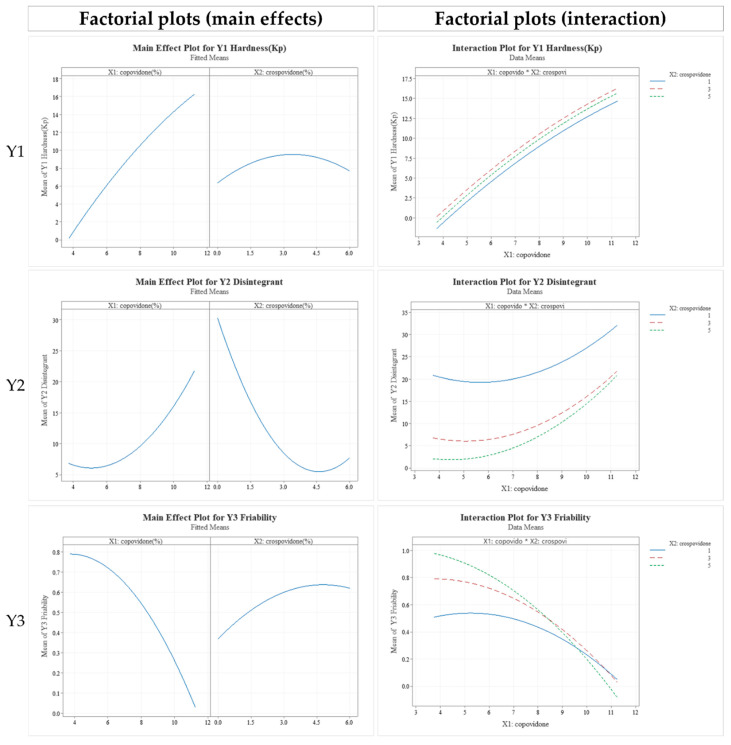

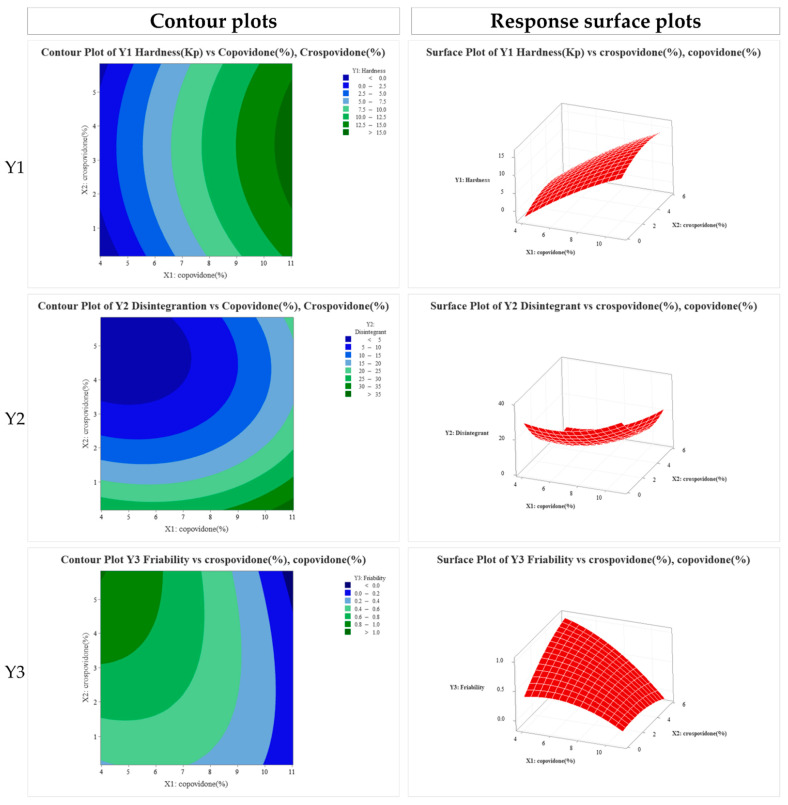

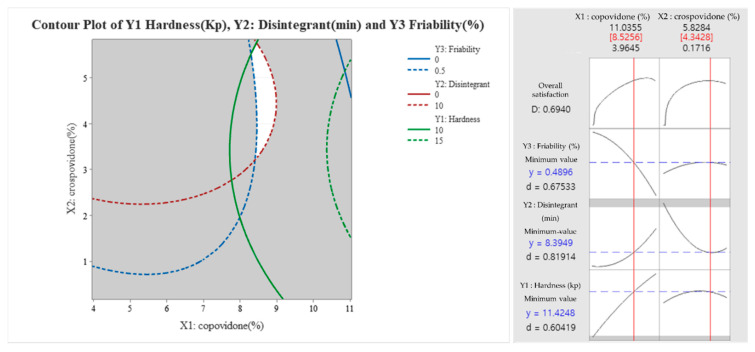
Effects of critical material attributes (CMAs) depicted by pareto chart and residual, factorial, contour, interaction, and response surface plots.

The central composite design of DoE was used to establish an appropriate management strategy for the CQAs of the outer layer and optimize its binding and disintegration properties. If the DoE is designed in a range that does not meet the quality attributes of the formulation, delivery of the main drug effect may be inadequate and excess drug might be used. Significant errors in the CQAs of the finished drug product could be propagated by manufacturing. Therefore, the ranges of the main response variables were 5.0–10.0% for copovidone and 1.0–5.0% for crospovidone based on preliminary experiments and standard CQA settings. Thirteen formulations were manufactured and evaluated to account for multidimensional binder and disintegrant combinations. The output variables were tablet hardness, disintegration time, and friability (Table 7). To justify these models, the residual terms at the experimental point were plotted on a normal probability. All residuals were collinear and evenly distributed within the 95.0% confidence level. Thus, all models were statistically significant. The effects of the binders and disintegrants on hardness, disintegration time, and friability were depicted in residual, main effect, interaction, contour, and response surface plots. The tablet CQAs were the target values for hardness, disintegration time, and friability, namely, ≥10 kp, ≤10 min, and <0.5%, respectively. The design space (DS) was derived from the overlapping contour plot (Figure 3). The DS of the response variable and influencing factor meeting established criteria were verified within the 95% confidence level. In Figure 3, the white area in the DS could serve as the actual design space. Optimization disclosed that the binder/disintegrant composition had hardness = 11 kp, friability = 0.489%, and disintegration time = 8 min. Sodium bicarbonate 800 mg, copovidone 8.5%, crospovidone 4.3%, and MgSO_4_ 0.2% were selected for the outer layer in subsequent experiments. The final composition of the RS dry-coated tablet was determined and a process study was conducted to identify the QTPP. The inner core was manufactured by wet granulation in the interest of content uniformity. Management of the related substances during granulation was the most important factor and continuous in-process control was performed. For the outer layer, granulation was difficult as the API sodium bicarbonate was not thermostable. Consequently, the outer layer was manufactured by direct compression. The final prepared RS dry-coated tablet was the most effective formulation tested as it could stabilize the rabeprazole sodium inner core by releasing the sodium bicarbonate antacid from the outer layer. Moreover, the double seal coating and HPMC coating of the inner core improved stability by preventing contact between APIs and protecting the inner core until the sodium bicarbonate could sufficiently neutralize the gastric acid. This formulation was optimized for the immediate release of rabeprazole sodium and met the objective of the present study. The final RS dry-coated tablet formulation was manufactured and evaluated in a good manufacturing practice (GMP)-certified facility in preparation for scale-up research. No significant problems were observed between the raw material mixing step and the production of the final formulation. Furthermore, tablet quality was sustained at a high level for a long time and the product had long-term physicochemical stability. The final formulation was a small, rapidly dissolving tablet facilitating administration, favoring patient compliance, and providing immediate drug release. 

### 3.5. In Vitro Dissolution Profile

**Figure 4:**
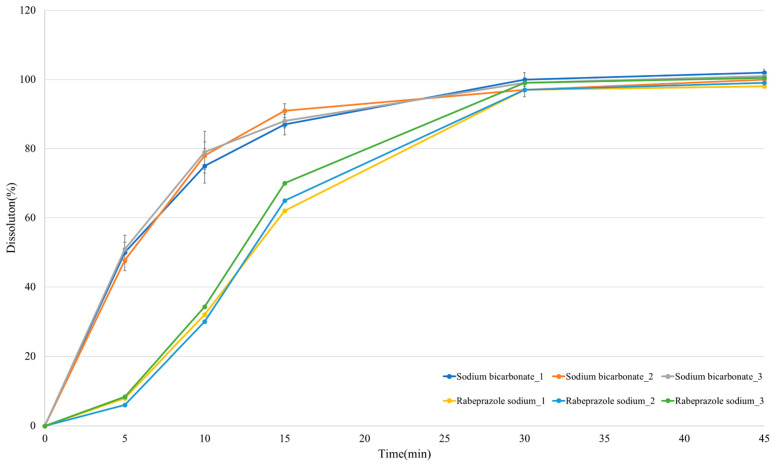
The in vitro dissolution profiles of RS dry-coated tablet at pH 8.0.

In solution, rabeprazole sodium was stable for ~26 h at pH 8.0. Eluate pH could be raised by neutralizing the sodium hydrogen carbonate in the outer layer. This reaction helps stabilize the rabeprazole sodium. Figure 4 shows the dissolution profiles for the rabeprazole sodium/sodium bicarbonate IR tablets. Sodium bicarbonate showed a dissolution rate of ≥90% within 15 min, whereas that of rabeprazole sodium was ≥80% within 30 min. The sodium bicarbonate in the outer layer was rapidly and completely released and raised the ambient pH. Rabeprazole sodium release began 15 min after sufficient neutralization was completed and most of the drug was released within 45 min. Hence, the formulation had a dissolution pattern enabling rapid absorption in the body as the rabeprazole sodium dissolved only after fast sodium bicarbonate release neutralized the gastric acid. Dissolution profiles confirm drug tablet disintegration and absorption patterns in the human body. The dry-coated tablet developed here was designed to disintegrate and support the absorption of rabeprazole sodium, which is unstable at the low stomach pH. Thus, evaluation and interpretation of the dissolution profile are important indices in experimental pK predictions. In vitro assays confirmed that the formulation design was suitable for the manufacture of IR tablets. Thence, the pharmacokinetic profile of the formulation should be investigated via an in vivo beagle pK test.

### 3.6. Stability Studies

Relative stability was determined and confirmed for RS dry-coated tablets and the reference drug (Pariet^®^) stored at room temperature for 12 months based on ICH guidelines Q1A and Q6A (Table 8). The average rabeprazole sodium content after 12 months of storage at RT was 99.8% for the RS dry-coated tablet and 98.3% for the reference drug. Both of these values were within the standard. The content uniformity test results were within 15% of the judgment value and met the standard. The dissolution rate was ≥80% within 30 min for both tablets. Therefore, there were no significant differences between tablets. After 12 months at RT, however, the total amounts of related substances were 1.01% for the dry-coated tablet and 1.68% for the reference drug. Thus, the total amount of related substances in the RS dry-coated tablet was approximately 0.6% lower than that in the reference drug. This difference demonstrates the enhanced stability of the dry-coated tablet compared to the conventional reference drug. Moreover, as rabeprazole sodium and sodium bicarbonate are incompatible, they should not interact under room temperature storage. After 12 months at RT, the average sodium bicarbonate content was 100.3%. The result of the formulation uniformity test was within 15% of the judgment value and suitable for the standard. The dissolution rate was ≥90% in 30 min. In all sodium bicarbonate test results, the values did not significantly differ between time zero and 12 months and the material was stable at RT. The Arrhenius equation in the Q10 expiration date calculation method predicted a 24-month shelf life at RT for the RS dry-coated tablet.

### 3.7. Pharmacokinetic Studies

**Figure 5:**
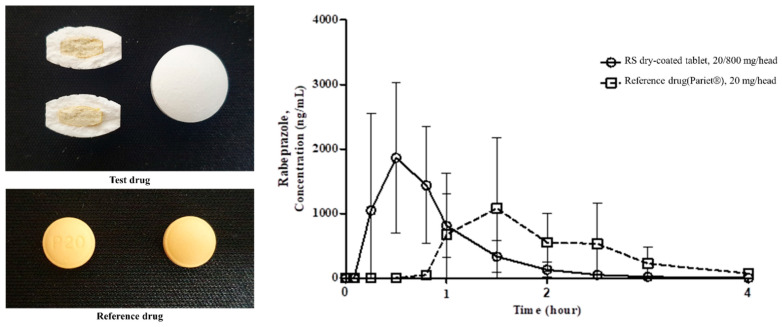
Mean (±SD) plasma concentrations-time profiles of rabeprazole following single oral administration of RS dry-coated tablet (20/800 mg) and Pariet tablet (20 mg) to beagles.

Figure 5 and Table 9 show that in vivo pharmacokinetic profiles were compared after oral administration of the RS dry-coated tablet and the reference drug to beagles. The AUC_last_ for the RS dry-coated tablet and the reference drug were 1614.0 ± 793.1 ng·h/mL and 1603.1 ± 864.6 ng·h/mL, respectively, and were considered equivalent. Their C_max_ were 2493.5 ± 1073.2 ng/mL and 1575.4 ± 991.4 ng/mL, respectively. Body exposure to the two drugs was similar; however, the half-lives were 0.4 h for the RS dry-coated tablet and 1.9 h for the reference drug. Meanwhile, the absorption velocity of the reference drug was slower than that of the RS dry-coated tablet. Hence, the former had a longer half-life. After oral administration of the RS dry-coated tablet and the reference drug to the dogs, systemic exposure to rabeprazole in terms of C_max_ and AUC_last_ was not statistically significant (*p* > 0.05). However, T_max_ for rabeprazole sodium was 0.5 ± 0.3 h and 1.5 ± 0.5 h, while that for the RS dry-coated tablet was ~3× faster than the reference drug. Moreover, the T_max_ for the RS dry-coated tablet was ≤Ft7× faster than that previously reported for enteric-coated rabeprazole sodium tablets. Hence, the RS dry-coated tablet resolved the problem of rabeprazole acid instability despite the rapid release of the drug in the stomach. Moreover, the RS dry-coated tablet had a markedly faster drug effect than enteric-coated rabeprazole tablets. Therefore, the RS dry-coated tablet developed herein could be an effective treatment for gastric acid-related diseases as it exerts therapeutic efficacy immediately after administration. This formulation prevents the decomposition of rabeprazole sodium by releasing sodium bicarbonate, which neutralizes gastric acid. Thence, the intact rabeprazole can be rapidly absorbed from the upper duodenum.

## 4. Conclusions

In the current study, we improved on conventional enteric rabeprazole sodium by adding the antacid sodium bicarbonate to the formulation. For this purpose, we applied the QbD method and developed a dry-coated tablet formulation comprising an IR rabeprazole sodium core and a protective sodium bicarbonate outer layer. The tablet was further stabilized by double coating the inner core with seal coating and HPMC coating, thereby preventing contact between the APIs. When the tablet reaches the stomach, the outer sodium bicarbonate layer will, therefore, protect the rabeprazole from the ambient stomach acid by neutralizing it and stabilizing the formulation both inside and outside the body. A DS was derived for the latter by using the central composite design of DoE, and the final formulation was optimized within the DS. The stability of the optimized formulation and the reference drug (Pariet^®^) was monitored at RT for 12 months. The average total impurity values were 0.36% at time zero, 0.55% at 6 months, and 1.03% at 12 months, which were 0.6% lower than those for the reference drug, demonstrating that the formulation had greater stability than the latter. Considering the total impurity and assay value after a 12-month storage period of the formulation, its shelf life predicted by the Arrhenius equation of the Q10 calculation method was confirmed to be > 24 months at RT. The RS dry-coated tablet dissolution profile further demonstrated that the prepared formulation enables the rapid dissolution and absorption of rabeprazole sodium in the stomach. Moreover, the beagle pKa study showed that the AUC_last_ for the manufactured tablets was 1614.0 ± 793.1 (ng·h/mL), which is comparable to that for the delayed release rabeprazole. However, the T_max_ of the former was ~3× faster than that of the latter. Thus, the RS dry-coated tablet developed herein could be an effective treatment for gastric acid-related diseases as it has therapeutic efficacy immediately after administration. Additionally, this formulation prevents the decomposition of rabeprazole sodium by releasing sodium bicarbonate to neutralize gastric acid. Thence, the intact rabeprazole can be rapidly absorbed from the upper duodenum. Collectively, the results of this study demonstrate that the tablets designed and tested here are physicochemically stable and rapidly absorb in the stomach. Therefore, they are expected to be highly efficacious and widely commercialized as a GERD treatment offering a faster onset time than the existing delayed-release PPI formulation. In the future, the technology used here could also be applied toward the formulation of other IR PPIs. Developed IR tablets can be used as a platform technology to overcome the technical and commercial limitations of various PPI drugs for patients with GERD requiring immediate therapeutic efficacy.

## Figures and Tables

**Table 1:** Quality target product profile (QTPP).

QTPP ^1^	Target	Risk	Justification
Indication	Reflux esophagitis	Yes	Inconsistent indications for patients taking this drug as a therapeutic drug may cause unnecessary drug misuse and side effects to the patient.
Dosage design	Immediate-release dry-coated tablets of less than 1100 mg	Yes	This drug is a dry-coated tablet formulation. Sodium bicarbonate in the outer layer is dissolved to act as an antacid in the stomach. After the antacid action is sufficiently completed, rabeprazole sodium in the inner core is released from the upper part of the small intestine to exert its medicinal effect. If the outer layer does not properly exert the antacid action, rabeprazole sodium can be decomposed in gastric acid, thereby reducing the patient’s therapeutic effect.
Route of administration	Oral administration	No	The oral route allows the administration of the highest drug dose and ensures high patient compliance.
Dosage strength	20 mg/800 mg once a day (Rabeprazole sodium 20 mg/Sodium bicarbonate 800 mg)	Yes	If the patient fails to take a certain dose, the patient’s treatment may deteriorate, and if the number of administration once a day increases to more than two times a day in consideration of long-term administration, the timing of administration may be missed.
Pharmacokinetics	The AUC is the same as the reference drug, but the T_max_ is about 3 times faster.	Yes	Maximum concentration (C_max_) and AUC can affect clinical trials such as safety and efficacy.
Stability and shelf life	Stable for at least 24 months at room temperature	Yes	If the quality characteristics of the drug are not suitable for the set period of use, the drug cannot be properly effective for the patients taking the drug.
Appearance	White circular shaped tablets	Yes	Changes in product appearance can lead to errors in the patient group’s selection of products for treatment.
Identification	Same equivalence requirement of peak retention time	No	When the purity of each active pharmaceutical ingredient is secured, there is no difficulty in identification.
Assay	90.0–110.0% of the label claim (as Rabeprazole sodium%, Sodium bicarbonate%)	Yes	If the content is high, it may cause side effects, and if the content is low it may affect the lack of efficacy.
Weight variation/ Content uniformity	Conforms to USP ^5^ <905> Uniformity of Dosage Units: 90.0–110.0% of labeled claim with AV ^6^: NMT 15.0; RSD: NMT 5.0%	Yes	Variability in content uniformity can affect adverse drug reactions and clinical response.
Dissolution	Rabeprazole sodium: NLT ^2^ 80% of labeled amount of drug is dissolved in 30 min in pH 8.0 buffer, Paddle speed: 75 rpm Sodium bicarbonate: NLT 90% of labeled amount of drug is dissolved in 30 min in water, Paddle speed: 75 rpm	Yes	The dissolution properties of the active pharmaceutical ingredients are important for bioavailability. (Especially, the salt of the main ingredient is important in evaluating the improvement of bioavailability by improving the solubility.)
Impurities	Unknown impurities: NMT ^3^ 0.2%, Total impurities: NMT 3.5% (As per ICH ^4^ Q3A and Q3B)	Yes	Related substances (unknown and total related substances) of the main active pharmaceutical ingredient must be managed.
Residual solvent	NMT 5000 ppm of ethanol	Yes	Ethanol, the residual solvent, must be managed.
Primary packaging	Packaging and container suitable for maintaining the physicochemical stability of pharmaceuticals	Yes	Packaging materials increase drug stability by protecting drugs from the surrounding environment. In addition, packaging materials in direct contact may react with drugs and promote their degradation.

^1^ QTPP: quality target product profile. ^2^ NLT: not less than. ^3^ NMT: not more than. ^4^ ICH: International Council for Harmonisation. ^5^ USP: united states pharmacopeia. ^6^ AV: acceptance value. Green: A wide range of acceptable risks. Yellow: An acceptable risk. Red: Unacceptable risk.

**Table 2:** Critical quality attributes (CQAs).

Quality Attributes of Dry Coated Tablet	Objective	CQA	Justification
Appearance	It should be in a shape and color for patients’ convenience in taking, and as a tablet, no defects should be observed.	Yes	Color, shape, and appearance are not directly linked to safety and effectiveness. However, in the case of sodium bicarbonate, which is used as the active pharmaceutical ingredient of the target tablet, the binding force is weak, and defects may be observed as a tablet. Therefore, this CQA should be studied through formulation research and process development.
	Tablet size for convenience in taking	No	To ensure patient adherence to therapy and to facilitate swallowing, the target of tablet size is minimized as long as no defects in the tablet are observed.
Identification	The main active pharmaceutical ingredient should be identified.	No	While the identification test is an important factor for safety and efficacy, this CQA can be effectively controlled by quality control systems and easily monitored in pharmaceuticals. Formulation studies and process parameters do not affect the identification test. Therefore, this CQA does not have to be discussed in formulation development and process development.
Assay	Rabeprazole sodium: 90~110% sodium bicarbonate: 90~110%	Yes	Variations in the assay can affect safety and effectiveness. Process variables can affect the content of the drug product. Therefore, the content should be evaluated through formulation research and process development.
Weight variation/ Content uniformity	Conforms to USP <905> Content uniformity: NMT 15.0%; RSD: NMT 5.0% Inner layer (Rabeprazole sodium): Content uniformity Outer layer (Sodium bicarbonate): Weight variation	Yes	– Variation in content uniformity can affect safety and effectiveness. Both formulation and process variables affect content uniformity. That is, this CQA should be evaluated through formulation research and process development. – Variations in weight variation can affect stability and effectiveness. Both formulation and process variables affect mass deviation. That is, this CQA should be evaluated through drug product research and process development.
Moisture content	Management in house spec according to stability test (less than 2.0%)	No	If the active ingredient is sensitive to moisture, stability, safety and efficacy may be affected. However, if the active ingredient is not sensitive to moisture or if appropriate packaging is used, the stability of the tablet will not be affected.
Impurities	Unknown impurities: NMT 0.2% Total impurities: NMT 3.5% (As per ICH Q3A and Q3B)	Yes	Degradation products may affect safety and should be controlled based on pharmacopeia or ICH requirements to limit exposure to patients. Limits for total related substances are based on the USP43-NF38. Formulation studies and process parameters can affect degradation products. Therefore, related substances must be evaluated during product and process development.
Residual solvent	USP <476> Option 1: NMT 5000 ppm of ethanol.	No	Residual solvent may affect safety, but it can be sufficiently controlled by the drying methods when manufacturing drugs or pharmaceuticals. Therefore, formulation studies and process variables are unlikely to have a significant impact on this CQA.
Dissolution	Rabeprazole sodium: NLT 80% of labeled amount of drug is dissolved in 45 min in pH 8.0 buffer, Paddle speed: 75 rpm Sodium bicarbonate: NLT 90% of labeled amount of drug is dissolved in 45 min in water, Paddle speed: 75 rpm	No	Failure to meet the dissolution conditions may affect bioavailability (efficacy). Formulation studies and process variables affect dissolution. However, rabeprazole sodium and sodium bicarbonate have very good solubility in most solvents, so they do not significantly affect the design of immediate-release.

Green: A wide range of acceptable risks. Yellow: An acceptable risk. Red: Unacceptable risk.

**Table 3:** Characteristics of rabeprazole sodium and sodium bicarbonate.

Rabeprazole Sodium			
Chemical structure	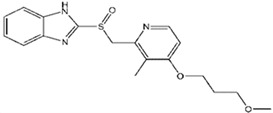	CAS. NO.	117976-90-6
		Chemical name	Rabeprazole sodium
		Formula	C_18_H_20_N_3_NaO_3_S
Mol. Mass	381.42 g/mol	Description	White powder
Melting point	140~141 °C	Solubility	10 mg/mL (Water)
Boiling point	603.9 °C		
PKa	pKa (Strongest Acidic): 9.35 pKa (Strongest Basic): 4.24	BCS Class	BCS III
Storage Condition	Airtight container, storage at room temperature		
Mechanism of action	It is a powerful proton pump inhibitor. Inhibits the secretion of gastric acid by inhibiting the parietal cell H+/K+ ATP pump.		
Pharmacokinetics	-Action onset time: within 1 h -Duration: 24 h -Absorption: Oral: well absorbed within 1 h; Food delays absorption by up to 4 h or more. -Protein binding rate: 96.3% -Metabolism: metabolized by CYP3A and 2C19 to inactive metabolites in the liver; CYP2C19 exhibits a genetic polymorphism that slows metabolism due to deficiency in some populations (subpopulations, Caucasian 3–5%, Asian 17-20%). -Bioavailability: Tablets: ~52% -Half-life (dose dependent): adolescents: ~0.55–1 h, adults: 1–2 h; 2–3 times higher in patients with mild to moderate hepatic impairment. -Time to reach maximum plasma concentration: Adolescents: Tablets: 3.3–4.1 h Adults: Tablets: 2–5 h; Capsule: 1–6.5 h -Excretion: urine (mainly 90% of metabolites of thioether carboxylic acid); The rest is feces		
**Sodium Bicarbonate**			
Chemical structure	* 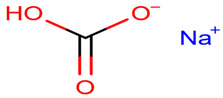 *	CAS. NO.	144-55-8
		Chemical name	Sodium bicarbonate
		Formula	NaHCO_3_
Mol. Mass	84.01 g/mol	Description	White, crystalline powder
Melting point	270 °C	Solubility	69 g/L (0 °C) 96 g/L (20 °C) 165 g/L (60 °C)
Boiling point	851 °C		
PKa	10.329 6.351 (carbonic acid)	BCS Class	BCSI
		USP	USP40-NF35
Storage Condition	Store in a tightly closed container. Store in a cool		
Mechanism of action	Separation produces bicarbonate ions that neutralize hydrogen ions and raise the pH of blood and urine. Neutralizing Additive (Dental Use): Increases the pH of Lidocaine and epinephrine solutions to improve tolerance and increase tissue absorption.		
Pharmacokinetics	-Onset time of action: oral: 15 min; Intravenous (IV): fast. -Duration: Oral: 1–3 h; Intravenous (IV): 8–10 min -Absorption: Oral: Well absorbed. -Excretion: urine (<1%)		
Characteristic	-As an antacid, it is used to improve symptoms caused by gastric ulcer, duodenal ulcer, gastritis, and excessive stomach acid. -As an alkalinizing agent, it is used for the purpose of reducing the acidity of blood or urine. -Widely used as a pH buffer.		

**Table 4:** Active pharmaceutical ingredient (API)-API compatibility studies (rabeprazole sodium and sodium bicarbonate) over four weeks.

Items	Initial (%)	4 Weeks (%)	
Rabeprazole sodium	0.06	Room Temperature	0.07
		Accelerated Condition	1.31
Rabeprazol sodium: Sodium bicarbonate 1:1 (*w*/*w*)	0.57	Room Temperature	0.79
		Accelerated Condition	2.17
Rabeprazol sodium: Sodium bicarbonate 20:800 (*w*/*w*)	3.41	Room Temperature	6.80
		Accelerated Condition	9.83

**Table:** (**a**)

CQAs ^1^	Rabeprazole Sodium	Sodium Bicarbonate	Diluent	Binder	Disintegrant	Anti-Adherent	Glidant	Lubricant
**Identification**	Low	Low	Low	High	Low	Low	Low	Low
**Assay**	Low	Low	Low	Low	High	Low	Low	Low
**Uniformity**	Low	Medium	Low	Medium	Low	Low	Low	Medium
**Impurities**	Low	Low	Low	Low	Low	Low	Low	Low
**Dissolution**	Low	Low	Low	High	High	Low	Low	Low

Green: A wide range of acceptable risks. Yellow: An acceptable risk. Red: Unacceptable risk.

**Table:** (**b**)

Functions	CMAs ^2^	Failure Mode (Critical Event)	Effect on CQAs with Respect to QTPP ^3^ (Justification of Failure Mode)	P ^6^	S ^7^	D ^8^	RPN ^9^
Physical property of API ^4^	Solid state form	Different PSD ^5^/ Different form	The solubility of the active pharmaceutical ingredient (API) may be affected, and the dissolution of the drug product is affected. Thus, this causes damage to bioavailability and efficacy.	1	2	2	4
Chemical property of API	Solubility	Different Salt/ Different form	May affect the dissolution of tablets. Thus, bioavailability and efficacy may be compromised.	1	1	2	2
	Chemical stability	Unstable	Decomposition products may be affected by dry heat/oxidation/hydrolysis/UV light, thus causing quality and safety damage.	1	1	2	2
Diluent	PSD	Uneven	It can affect the flow properties of blending and can affect the content uniformity. Thus, quality/safety may be compromised.	1	1	2	2
	Moisture Content	High	May affect the impurity profile. Thus, this causes damage to safety.	3	2	2	12
Binding solution (Inner layer)	Volume of binding solution	Higher than optimum	Produces hard granules, which can affect disintegration and dissolution time. Thus, bioavailability and efficacy may be compromised.	3	2	1	6
		Lower than optimum	Loose, fragile granules can produce tablets of weaker hardness (fast disintegration). Thus, bioavailability and efficacy may be compromised.	3	2	1	6
Binder (Outer layer)	Concentration of binder	Higher than optimum	Delayed disintegration and dissolution time of tablets. Thus, bioavailability and efficacy may be compromised.	4	5	3	60
		Lower than optimum	The friability of the tablet is high, and the desired dissolution pattern cannot be obtained. Thus, bioavailability and efficacy may be compromised.	4	4	3	48
Disintegrant	Concentration of disintegrant	Higher than optimum	The desired dissolution pattern cannot be obtained, and the hardness of the tablet may be affected. Thus, bioavailability and efficacy may be compromised.	4	4	3	48
		Lower than optimum	The desired dissolution pattern cannot be obtained. Thus, bioavailability and efficacy may be compromised.	4	3	3	36
Anti-adherent	Concentration of Anti-adherent	Lower than optimum	It may be difficult to discharge tablets from tooling. The excipient can be stuck on the surface of the filling die. Thus, product quality may be compromised.	3	3	2	18
Glidant	Concentration of glidant	Lower than optimum	By reducing the friction in the particles, it may affect the flowability of granules or powders such as die friction. May affect content uniformity. Therefore, content uniformity and product quality may be compromised.	2	2	2	8
Lubricant	Concentration of Lubricant	Higher than optimum	Hydrophobic lubricants can be coated on the surface of drug particles, which can delay dissolution. Thus, efficacy may be compromised.	3	3	3	27
		Lower than optimum	The powder can stick to the surface of tooling/punch and cause picking. Thus, product quality may be compromised.	3	3	3	27

^1^ CQAs: critical quality attributes. ^2^ CMAs: critical material attributes. ^3^ QTPP: quality target product profile. ^4^ API: active pharmaceutical ingredient. ^5^ PSD: particle size distribution. ^6^ P: probability. ^7^ S: severity. ^8^ D: detect ability. ^9^ RPN: risk priority number; if the total RPN is more than 30 scores (marked red), in order to be prepared for risks, major actions such as DOE must be performed.

**Table:** (**a**)

CQAs	Screening	Blending	Granulation	Drying	Milling	Blending and Lubrication	Compression
**Identification**	Low	Low	Low	Medium	Low	Low	Medium
**Assay**	Low	Low	Medium	Low	Low	Low	Medium
**Uniformity**	Low	Low	Low	Low	Low	Low	Low
**Impurities**	Low	Low	Low	Medium	Low	Low	Low
**Dissolution**	Low	Low	Low	Low	Low	Medium	Medium

Green: A wide range of acceptable risks. Yellow: An acceptable risk. Red: Unacceptable risk.

**Table:** (**b**)

Functions	CPPs ^1^	Failure Mode (Critical Event)	Effect on CQAs ^2^ with Respect to QTPP^3^ (Justification of Failure Mode)	P	S	D	RPN
Screening	Sifting	Larger than optimum sieve size	Uneven particle size mixture could cause content non-uniformity. Thus, quality and safety may be compromised.	1	1	1	1
Blending	Mixing rate (Rpm and Time)	Lower mixingspeed and shorter time	Insufficient total number of revolutions leads to the inhomogeneity of the mixture. Thus, bioavailability and efficacy may be compromised.	1	2	1	2
Granulation	Impeller/Mixer speed	Higher mixing speed and longer time	Production of large granules (agglomerate/lumps) increases the elution time of tablets. Thus, bioavailability and efficacy may be compromised.	3	2	2	12
	Chopper/Granulator speed	Lower mixing speed and shorter time	Production of large granules (agglomerate/lumps) increases the elution time of tablets. Thus, bioavailability and efficacy may be compromised.	3	2	2	12
	Granulation time	Longer than optimum time	Production of large granules (agglomerate/lumps) increases the elution time of tablets. Thus, bioavailability and efficacy may be compromised.	3	2	2	12
Drying	Inlet temperature	Lower than optimum temperature	If the temperature is lower than the optimum temperature, the solution is not dried well, and the residual solvent affects the physical aspect of the tablet.	2	2	3	12
		Higher product temperature	Degradation and impurities profile may be affected. Thus, safety and efficacy may be compromised	3	2	3	18
Milling	Mill speed	Higher than optimum speed	Poor flow and non-uniformity can occur due to the generation of fine powder.	1	1	2	2
	Mill screen size	Larger than optimum screen size	Uneven PSD causes inhomogeneity. Larger particles increase the dissolution time. Thus, efficacy may be compromised.	1	1	2	2
Blending and lubrication	Blending rate (RPM and Time)	Higher than optimum speed and longer time	Dissolution time may increase. Thus, efficacy may be compromised.	1	2	2	4
Compression	Speed of turret and feeder	Higher than optimum speed	Lamination and weight variation can be observed = It affects content uniformity, disintegration time, and dissolution. Thus, efficacy may be compromised.	4	1	6	24
	Compression force (Pre-compression and compression)	Higher than optimum force	The appearance and hardness of tablets may be affected. Disintegration and dissolution profiles may be affected. Thus, efficacy may be compromised.	3	2	2	12
Coating	Speed of coating pan	Higher than optimum speed	If the speed of the coating pan is high, it causes damage to the tablet. Thus, bioavailability and efficacy may be compromised.	3	1	1	3

^1^ CPPs: critical process parameters. ^2^ CQAs: critical quality attributes. ^3^ QTPP: quality target product profile. ^4^ RPN: risk priority number.

**Table 7:** Critical material attributes (CMAs) affecting critical quality attributes (CQAs).

Run	CMAs		CQAs		
	X1: Copovidone (%)	X2: Crospovidone (%)	Y1: Hardness (kp)	Y2: Disintegration (min)	Y3: Friability (%)
1	5.0	1.0	1.7	19.3	0.8
2	10.0	1.0	10.8	28.3	0.2
3	5.0	5.0	2.3	4.0	1.3
4	10.0	5.0	11.6	18.0	0.3
5	3.9	3.0	0.9	6.0	0.4
6	11.0	3.0	18.3	18.0	0.1
7	7.5	0.2	7.8	28.3	0.3
8	7.5	5.8	9.3	4.0	0.3
9	7.5	3.0	9.5	8.5	0.6
10	7.5	3.0	9.5	8.5	0.6
11	7.5	3.0	9.5	8.5	0.6
12	7.5	3.0	9.5	8.5	0.6
13	7.5	3.0	9.5	8.5	0.6

**Table 8:** Twelve-month RS dry-coated tablet stability studies.

APIs	Stability	Criterion	Drugs	Initial	6 Month	12 Month
Rabeprazole sodium	Assay	90–110%	Test drug	100.8 ± 1.2%	102.7 ± 0.5%	99.8 ± 1.3%
			Reference drug	100.2 ± 0.8%	98.9 ± 1.3%	98.7 ± 1.7%
	Content uniformity	NMT 15%	Test drug	3.12 ± 0.1%	3.08 ± 2.5%	3.30 ± 0.3%
			Reference drug	5.27 ± 1.2%	5.64 ± 1.1%	5.71 ± 0.6%
	Dissolution	NLT 80%	Test drug	91.2 ± 2.4%	92.8 ± 0.8%	90.1 ± 1.1%
			Reference drug	90.4 ± 1.8%	92.1 ± 1.4%	91.4 ± 0.9%
	Total impurities	NMT 3.5%	Test drug	0.32 ± 0.2%	0.55 ± 0.3%	1.01 ± 1.2%
			Reference drug	0.38 ± 0.1%	0.72 ± 0.1%	1.28 ± 0.7%
Sodium bicarbonate	Assay	90–110%	Test drug	101.7 ± 1.3%	102.2 ± 2.1%	100.3 ± 1.7%
	Content uniformity	NMT 15%	Test drug	4.14 ± 0.1%	3.98 ± 0.4%	4.27 ± 0.7%
	Dissolution	NLT 90%	Test drug	102.3 ± 0.2%	101.4 ± 0.7%	100.6 ± 1.4%

NMT: not more than. NLT: not less than. Mean ± S.D. (*n* = 6).

**Table 9:** Mean (±SD) pharmacokinetic parameters of rabeprazole following single oral administration of test drug, 20 mg and reference drug, 20 mg to beagles.

PK Parameter	RS Dry-Doated Tablet	Reference Drug
	20 mg/head	20 mg/head
Cmax ^1^ (ng/mL)	2493.5 ± 1073.2	1575.4 ± 991.4
Tmax ^2^ (hr) ^1)^	0.5 (0.3–0.8)	1.5 (1–2.5)
AUClast ^3^ (ng·hr/mL)	1614.0 ± 793.1	1603.1 ± 864.6
AUCinf (ng·hr/mL)	1620.2 ± 793.8	1782.7 ± 886.8
T1/2 (hr)	0.4	1.9 ± 2.6
CL/F ^4^ (mL·hr/kg)	15,930.4 ± 10,034.3	15,822.5 ± 13,023.2
Vd/F ^5^ (mL/kg)	8182.0 ± 5617.7	47,350.3 ± 56,108.4
Rsq_adjusted	1.0	0.7 ± 0.5
%AUCexp (%)	0.5 ± 0.5	10.1 ± 19.6

^1^ C_max_: maximum plasma concentration. ^2^ T_max_: time to C_max_. presented as median (Min-Max). ^3^ AUC_t_: area under the plasma concentration-time curve to the last sampling time. ^4^ CL/F: volume of distribution. ^5^ Vd/F: clearance.

## Data Availability

The data presented in this study are included in this published article.
